# Large‐scale movement patterns in a social vulture are influenced by seasonality, sex, and breeding region

**DOI:** 10.1002/ece3.9817

**Published:** 2023-02-08

**Authors:** Jon Morant, Eneko Arrondo, José Antonio Sánchez‐Zapata, José Antonio Donázar, Ainara Cortés‐Avizanda, Manuel De La Riva, Guillermo Blanco, Félix Martínez, Juan Oltra, Martina Carrete, Antoni Margalida, Pilar Oliva‐Vidal, José Maria Martínez, David Serrano, Juan Manuel Pérez‐García

**Affiliations:** ^1^ Department of Applied Biology Miguel Hernández University of Elche Elche Spain; ^2^ Centro de Investigación e Innovación Agroalimentaria y Agroambiental (CIAGRO‐UMH) Miguel Hernández University of Elche Orihuela Spain; ^3^ Departament of Conservation Biology Estación Biológica de Doñana (CSIC) Sevilla Spain; ^4^ Department of Plant Biology and Ecology, Faculty of Biology University of Seville Seville Spain; ^5^ Department of Evolutionary Ecology Museo Nacional de Ciencias Naturales (CSIC) Madrid Spain; ^6^ Escuela Internacional de Doctorado Universidad Rey Juan Carlos (URJC) Madrid Spain; ^7^ Department of Physical, Chemical and Natural Systems Universidad Pablo de Olavide Sevilla Spain; ^8^ Pyrenean Institute of Ecology (CSIC) Jaca Spain; ^9^ Institute for Game and Wildlife Research IREC (CSIC‐UCLM) Ciudad Real Spain; ^10^ Department of Animal Science, Faculty of Life Sciences and Engineering University of Lleida Lleida Spain; ^11^ Departamento Medio Ambiente, Gobierno de Aragón Subdirección General de Desarrollo Rural y Sostenibilidad Huesca Spain

**Keywords:** griffon vulture, home‐range, scavenger, site fidelity, spatial segregation

## Abstract

Quantifying space use and segregation, as well as the extrinsic and intrinsic factors affecting them, is crucial to increase our knowledge of species‐specific movement ecology and to design effective management and conservation measures. This is particularly relevant in the case of species that are highly mobile and dependent on sparse and unpredictable trophic resources, such as vultures. Here, we used the GPS‐tagged data of 127 adult Griffon Vultures *Gyps fulvus* captured at five different breeding regions in Spain to describe the movement patterns (home‐range size and fidelity, and monthly cumulative distance). We also examined how individual sex, season, and breeding region determined the cumulative distance traveled and the size and overlap between consecutive monthly home‐ranges. Overall, Griffon Vultures exhibited very large annual home‐range sizes of 5027 ± 2123 km^2^, mean monthly cumulative distances of 1776 ± 1497 km, and showed a monthly home‐range fidelity of 67.8 ± 25.5%. However, individuals from northern breeding regions showed smaller home‐ranges and traveled shorter monthly distances than those from southern ones. In all cases, home‐ranges were larger in spring and summer than in winter and autumn, which could be related to difference in flying conditions and food requirements associated with reproduction. Moreover, females showed larger home‐ranges and less monthly fidelity than males, indicating that the latter tended to use the similar areas throughout the year. Overall, our results indicate that both extrinsic and intrinsic factors modulate the home‐range of the Griffon Vulture and that spatial segregation depends on sex and season at the individual level, without relevant differences between breeding regions in individual site fidelity. These results have important implications for conservation, such as identifying key threat factors necessary to improve management actions and policy decisions.

## Graphical abstract

Griffon vulture home‐range sizes and home‐range fidelity are affected by seasonality, sex, and breeding region. Vultures tend to move more during spring and summer, and females showed higher home‐range sizes than males, but lower home‐range fidelity. Overall, vultures showed slow differences between breeding regions in home‐range size and fidelity.

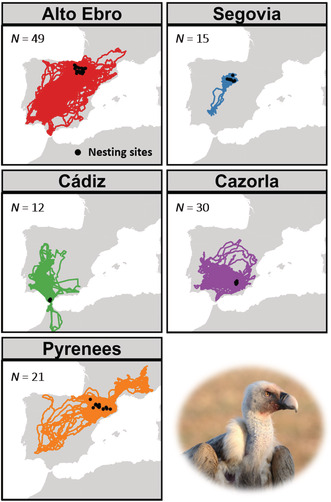

## Resumen

Cuantificar el uso y la segregación del espacio, así como los factores extrínsecos e intrínsecos que los afectan, es crucial para aumentar nuestro conocimiento de la ecología de movimientos de cada especie y diseñar medidas eficaces de gestión y conservación. Esto es especialmente relevante en el caso de especies con gran movilidad y dependientes de recursos tróficos escasos e impredecibles, como son los buitres. En este trabajo se utilizaron datos GPS de 127 buitres leonados *Gyps fulvus* adultos capturados en cinco regiones de cría diferentes en España para describir los patrones de movimiento (tamaño y fidelidad del área de campeo y distancia acumulada mensual). También examinamos cómo el sexo, la estación del año y la región de cría determinaban la distancia acumulada recorrida y el tamaño y solapamiento entre áreas de campeo mensuales consecutivas. En conjunto, los buitres leonados mostraron un área de campeo anual muy extensa de 5027 ± 2123 km^2^, una distancia acumulada mensual media de 1776 ± 1497 km y una fidelidad mensual al área de campeo del 67.8 ± 25.5%. Sin embargo, los individuos de las regiones de cría más septentrionales mostraron áreas de campeo más pequeñas y recorrieron distancias mensuales más cortas que los de las más meridionales. En todos los casos, las áreas de campeo fueron mayores en primavera y verano que en otoño e invierno, lo que podría estar relacionado con las diferencias en las condiciones de vuelo y las necesidades tróficas asociadas a la reproducción. Además, las hembras mostraron mayores áreas de campeo y menor fidelidad mensual que los machos, lo que indica que estos últimos tienden a utilizar zonas similares durante todo el año. En conjunto, nuestros resultados indican que tanto los factores extrínsecos como los intrínsecos modulan el área de campeo del buitre leonado y que la segregación espacial depende del sexo y de la estación a nivel individual, sin que existan diferencias relevantes entre las regiones de cría en cuanto a la fidelidad individual al lugar. Estos resultados podrían tener importantes implicaciones para la conservación, como la identificación de los principales factores necesarios para mejorar las medidas de gestión y las decisiones políticas.

## 1 INTRODUCTION

Animal movements are a consequence of an organism's internal state (e.g., sex, age, breeding stage) and environmental factors (e.g., food availability or weather), and can affect individual fitness and ecological processes at local and global scales (Hansson et al., 2014). Individual movements are also influenced by inter and intraspecific relationships (e.g., competition), which may lead to spatial compartmentalization and the maintenance of population‐specific movement patterns over time (Nathan et al., 2008). Deciphering how these factors modulate individual movements and how the latter are compartmentalized in space and time is essential to understanding population dynamics (Costa‐Pereira et al., 2022) and identifying priority areas for conservation and management (Katzner & Arlettaz, 2020).

Home‐range size and cumulative distance traveled are key elements in the study of animal movement ecology (Kie et al., 2010; Shaw, 2020; Thaker et al., 2019; Tucker et al., 2018), defining foraging patterns at the individual and population‐level, and assessing their stability over time (e.g., Shaffer et al., 2017). For example, investigating variation in home‐range size and cumulative distance traveled may reveal that certain individuals behave as central‐place foragers during only a specific period of their life cycle (e.g., the breeding season; Carrete & Donázar, 2005; Delgado‐González et al., 2022). Similarly, the study of home‐range overlap allows the analysis of attraction or repulsion relationships that may affect space use, for example, to avoid competition for resources (Bolnick et al., 2003; Cecere et al., 2018; Winner et al., 2018). This information could be used to explicitly map intra and interspecific meeting sites and prioritize high‐quality habitats for communal roost or feeding hotspots (Cortés‐Avizanda et al., 2014; Kane et al., 2015).

Vultures from the *Gyps* genus (which includes seven species) are among the largest flying birds, showing high sociality and covering large areas in search of ephemeral and unpredictable resources such as carrion (e.g., 162,824 km^2^ annually on average in the case of Cape Vultures, *Gyps coprotheres*; Jobson et al., 2021). The Eurasian Griffon Vulture *Gyps fulvus* is a monomorphic social species that breeds colonially (Almaraz et al., 2022; Donázar, 1993; Harel et al., 2017; Zuberogoitia et al., 2018). The breeding period of the species expands from early December (when first copulates occurs) to late August, when fledglings fly from the nest (Donázar, 1993; Zuberogoitia et al., 2018). Individuals forage over vast areas to satisfy their energetic requirements (e.g., ranging annually from 1560 to 4233 km^2^, Fluhr et al., 2021; Monsarrat et al., 2013; Nathan et al., 2012; Xirouchakis et al., 2021), frequently congregating around both wild and domestic ungulate carcasses (Cortés‐Avizanda et al., 2010, 2012 but see also Delgado‐González et al., 2022). Although information exists on Griffon vulture movement ranges (Arkumarev et al., 2021; Arrondo et al., 2018; Arrondo, Sanz‐Aguilar, et al., 2020; Fluhr et al., 2021; García‐Ripollés et al., 2011; Harel et al., 2017; Spiegel et al., 2013, 2015; Xirouchakis et al., 2021; Xirouchakis & Mylonas, 2007; Zuberogoitia et al., 2013), virtually nothing is known about the spatio‐temporal variation in the movement patterns of adult individuals, or about the factors (e.g., sex, breeding region) governing the spatial ecology and home‐range fidelity of this species from a mechanistic perspective.

In this paper, we gather movement data from 127 GPS‐tagged adult Griffon Vultures captured in five breeding regions of peninsular Spain, the largest vulture population in Western Palearctic encompassing up to 37,000 breeding pairs (90% of all European populations) (Del Moral & Molina, 2018). Our main aim is to assess the effect of individual and environmental factors on movements and spatial use indicators. Specifically, our objectives are: (1) to estimate annual and monthly home‐range sizes, monthly cumulative distances traveled, and monthly home‐range site fidelity; and (2) to investigate the effect of season, sex and breeding regions on individual monthly home‐range size, site fidelity, and cumulative distance. We hypothesize that adult Griffon Vultures, being a large monomorphic colonial species, will exhibit large home‐ranges and will travel long distances to fulfill their requirements (mainly food), especially during the autumn and winter, when food availability is the lowest (Margalida et al., 2018; Spiegel et al., 2013). We also predict that the fidelity of monthly home‐ranges should be similar between sexes due to the lack of dimorphism, but would differ between seasons, as foraging constraints are more likely during the breeding period (see Carrete & Donázar, 2005). Finally, we expect differences between breeding regions due to differences in resource availability (Morant et al., 2022).

## 2 METHODS

### 2.1 Capture and tagging of vultures

**FIGURE 1:**
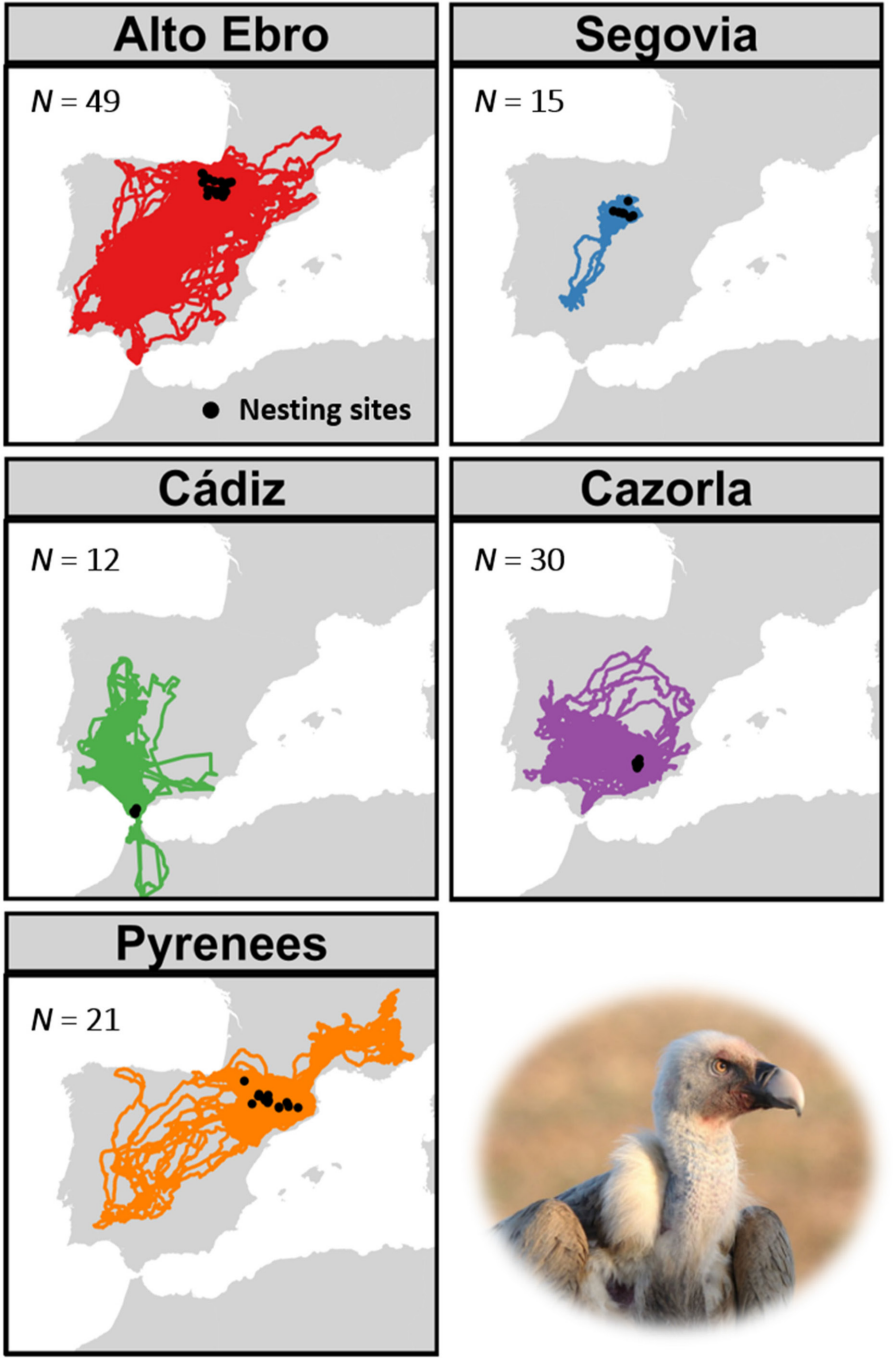
Movements and nest locations of 127 GPS‐tagged adult Griffon Vultures in northern (Alto Ebro and Pyrenees), central (Segovia), and southern (Cádiz and Cazorla) Spain. *N* represents the number of tagged vultures for each breeding region. Photo credit: Manuel de la Riva.

From 2014 to 2022, we captured 127 adult Griffon Vultures (43 males and 84 females) in five breeding regions distributed across northern (Alto Ebro: 49 individuals, Pyrenees: 21 individuals), central (Segovia: 15 individuals), and southern Spain (Cádiz: 12 individuals and Cazorla: 30 individuals) (see Figure 1). Breeding regions were delimited according to the proximity between nesting sites and the biogeographic characteristics of each area where nests are located. Birds were trapped using remotely activated cannon nets and cage traps baited with livestock carcasses. Individuals were tagged with yellow or blue plastic alphanumeric and metal rings and equipped with solar‐powered GPS/GSM transmitters (Ecotone https://ecotone‐telemetry.com/en, Ornitela https://www.ornitela.com/, and e‐Obs https://e‐obs.de/). Devices were attached using a Teflon tape backpack harness. The total weight of the transmitters and rings did not exceed 64 g, which represented less than 3% of the body weight of the individuals (Bodey et al., 2018). The age of individuals was estimated from plumage molt and other external features such as the color of the culmen and the eyes (Donázar, 1993; Zuberogoitia et al., 2013), while sex was determined using molecular sexing techniques from body feather samples (Fridolfsson & Ellegren, 1999).

Tracking devices were programmed to record fixes (i.e., GPS positions) at 5–10‐min intervals from 1 h before sunrise to 1 h after sunset (see Table 1 for details of the tracking devices and sampling frequency). GPS data were incorporated into the Movebank online data repository (www.movebank.org). Data were standardized by resampling the GPS fixes to 15 min for each individual to homogenize our dataset. Vultures were tracked on average 1040 ± 809 days with a mean number of fixes per individual of 41,335 ± 40,493.

**TABLE 1:** Annual and monthly home‐range size (in km^2^; estimated at 95% KDE), and cumulative distance traveled (in km) of 127 adult Griffon vultures tagged with GPS in Spain.

	Home‐range (km^2^)	Cumulative distance traveled (km)
Annual	5027 ± 2123 (1981–9863)	15,090 ± 11,256 (219–39,298)
Monthly	4889 ± 1753 (1908–6822)	1776 ± 1497 (108–7172)

*Note*: All values area shown with mean (± SD) and their range (minimum and maximum values) in brackets.

### 2.2 Estimation of home‐range, cumulative distance traveled, and site fidelity

We estimated annual and monthly home‐ranges using the 95% kernel density estimator contours (KDE) and the cumulative distance traveled for each tagged individual using the “amt” package (Signer et al., 2019). We selected KDE function instead of AKDE since this had no qualitative improvement to our results and notably increased computational time compared to KDE function (see for instance Silva et al., 2022). Cumulative distance traveled per month was measured as the total length of each track (in km) (Edelhoff et al., 2016). Individuals with less than ten fixes per day were discarded (*n* = 4 cases).

We measured the individual home‐range fidelity by calculating the percentage of overlap between consecutive monthly home‐range (Fieberg & Kochanny, 2005). We selected the 95% KDE as an estimator of home‐range to evaluate differences in foraging areas. The percentage of overlap was quantified using the Bhattacharyya coefficient, which ranges between 0 (complete segregation) and 1 (perfect overlapping; Fieberg & Kochanny, 2005; Winner et al., 2018).

### 2.3 Data analysis

We analyzed the effect of sex, season, and breeding regions on the monthly home‐range sizes, cumulative monthly distances traveled (normal error distribution, identity link function), and percentage of monthly overlap (beta error distribution, identity link function) using generalized linear mixed models (GLMMs; glmmTMB package, Brooks et al., 2017). Sex, season, and breeding region were included as fixed factors, while individual identity and year were included as random terms in the models to avoid pseudo replication.

We did not divide the period into breeding stages since we could not rule that all individuals were breeding each monitoring year and, therefore, followed the same breeding cycle periods. Instead, we here referred to the season as the division of the year based on changes in weather, ecology, and the number of daylight hours in a given region. In the case of the breeding region, we regard this variable, not only as an indicator of differences in the periods suitable for soaring (mediated by photoperiod; see Scacco et al., 2019) but also as a potential proxy of differences in the food available in the field (e.g., some regions encompass much more carrion than others in absolute numbers; Morant et al., 2022) and population differences in the levels of exposure to known threats (i.e., risk of collision, electrocution or poisoning; Arrondo, Sanz‐Aguilar, et al., 2020).

Finally, we did not consider the interaction between the aforementioned variables due to (1) the complexity of the understanding that is implicit to them (e.g., sex differences in movement associated with particular regional conditions) and (2) to avoid speculations without any empirical foundations in the interpretation of the results.

Models were compared using the Akaike Information Criterion corrected for small sample sizes (AICc; Burnham & Anderson, 2002). The best model was the one with the lowest AICc value. All models with a difference of ΔAICc < 2 were considered alternatives (Burnham & Anderson, 2002). For the best model, homogeneity of variance and normality of residuals was inspected by using the “ggresid” package to check the goodness‐of‐fit of our best models (Goode & Rey, 2019). We estimated the variance explained by the fixed and random factors by using the “performance” package (Lüdecke et al., 2021), while differences between their levels were assessed through Tukey's post hoc tests using the “emmeans” package (Lenth, 2022). All tests were two‐tailed, statistical significance was set at α =0.05, and all results were shown as mean ± standard deviation. Results of the Tukey's post hoc tests included the marginal means and its standard error. Spatial and statistical analyses were done in R version 4.0.3 (R Core Team, 2021).

## 3 RESULTS

**FIGURE 2:**
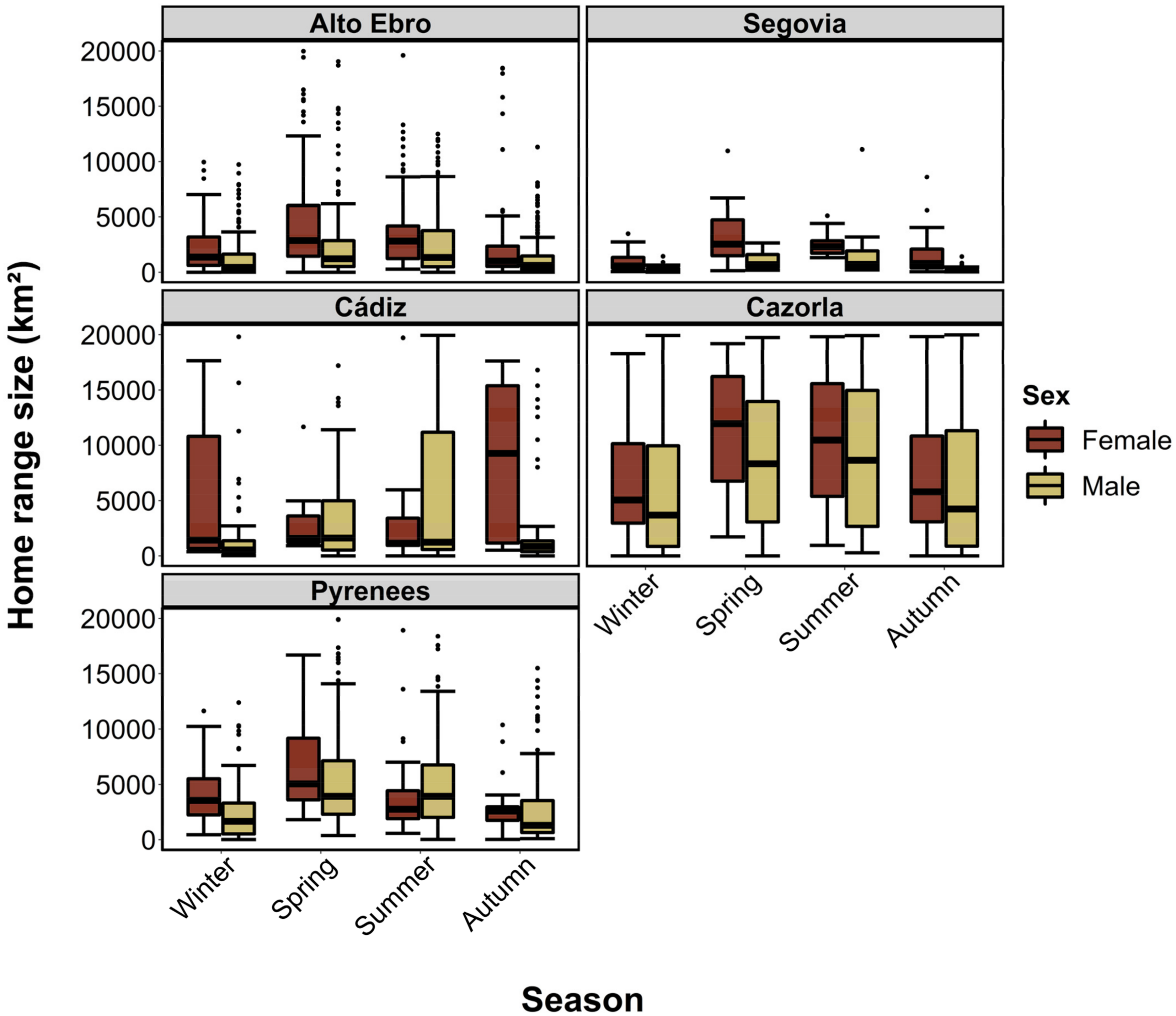
Mean monthly home‐range size (in km^2^, based on 95% KDE ± SD) of 127 adult Griffon Vultures (43 females and 84 males) tagged in northern (Alto Ebro and Pyrenees), central (Segovia), and southern (Cádiz and Cazorla) Spain. The standard deviation is shown as error bars.

Griffon Vultures exploited very large annual home‐range sizes (ca. 5000 km^2^), showing average monthly cumulative distances traveled of 1776 km (Table 1), and a monthly home‐range fidelity of 67.77 ± 25.05%. Monthly home‐ranges differed between sexes, seasons, and breeding regions (Tables 2 and 3; Figure 2). Males showed a smaller monthly home‐range size than females (Table 3). Birds also showed larger home‐ranges during spring and summer compared to autumn and winter (Table S2; Figure 2). Individuals from southern breeding regions (Cazorla, Cádiz), and central Pyrenees showed, on average, larger home‐ranges than those of central (Segovia) and northern breeding regions (Alto Ebro) (Table S2 and Figure 2).

**TABLE 2:** Models obtained to assess the effects of individual sex, season, and population on the movement and spatial segregation patterns of 127 GPS‐tagged adult Griffon Vultures in Spain.

	Model	*k*	AIC_c_	ΔAIC_c_	AIC_cw_	*R* ^2^ fixed	*R* ^2^ random
Movement patterns							
Home‐range size (km^2^)	**Season + sex + breeding region**	**6**	**59582.1**	**0.00**	**0.993**	**28.18**	**20.61**
	Breeding region + season	5	59612.7	30.56	0.007		
	Breeding region + sex	5	59849.0	266.88	0.000		
	Season + sex	5	59862.2	280.05	0.000		
	Season	4	59877.6	295.45	0.000		
	Breeding region	4	59877.7	295.58	0.000		
	Sex	4	60132.9	550.79	0.000		
	Null	3	60147.5	565.39	0.000		
Cumulative distance (km)	**Season + sex + breeding region**	**6**	**67835.8**	**0.00**	**0.988**	**33.29**	**19.25**
	Season + breeding region	5	67844.6	8.74	0.012		
	Season + sex	5	67941.3	105.48	0.000		
	Season	4	67952.4	116.59	0.000		
	Breeding region + sex	5	69656.5	1820.6	0.000		
	Breeding region	4	69665.9	1830.	0.000		
	Sex	4	69771.4	1935.6	0.000		
	Null	3	69784.4	1948.5	0.000		
Spatial segregation							
Monthly home‐range fidelity (%)	**Season + sex + breeding region**	6	**−1415.9**	**0.00**	**0.998**	**0.842**	**1.974**
	Season + breeding region	5	−1392.5	23.45	0.002		
	Season + sex	5	−1371.2	44.73	0.000		
	Season	4	−1353.0	62.90	0.000		
	Breeding region + sex	5	−1230.5	185.46	0.000		
	Breeding region	4	−1209.2	206.70	0.000		
	Sex	4	−1189.4	226.54	0.000		
	Null	3	−1171.3	244.57	0.000		

*Note*: The null model was included in our set of models. For each best model, variability (as a percentage) explained by the fixed and random predictors (*R* ^2^) are shown. The best models (ΔAICc < 2) are highlighted in bold.

Abbreviations: AICc: Akaike Information Criterion corrected for small sample sizes; k: number of parameters; w: Akaike weight; ΔAICc: difference between the AICc of model i and that of the best model (i.e., the model with the lowest AICc).

**TABLE 3:** Estimates for fixed terms of the best models of monthly home‐range size, cumulative distance traveled per month and monthly home‐range fidelity for 127 GPS‐tagged adult Griffon Vultures in Spain.

Response variable	Predictors	Estimate ± SE	*z* value	*p*‐value
Monthly home‐range size	Season (spring)	2250.6 ± 193	11.660	**<.001**
	Season (summer)	2195.7 ± 195.6	11.225	**<.001**
	Season (autumn)	204 ± 193.9	1.053	.292
	Sex (male)	−1294.5 ± 291	−4.449	**<.001**
	Breeding region (Segovia)	−899.8 ± 586.8	−1.533	.125
	Breeding region (Cádiz)	1670.7 ± 507.2	3.294	**.001**
	Breeding region (Cazorla)	5750 ± 319.8	17.980	**<.001**
	Breeding region (Pyrenees)	1920.3 ± 397.9	4.827	**<.001**
Cumulative distance	Season (spring)	1393.69 ± 47.05	29.622	**<.001**
	Season (summer)	1925.70 ± 48.43	39.763	**<.001**
	Season (autumn)	162.46 ± 49.79	3.263	**.001**
	Sex (male)	41.15 ± 73.88	0.557	.577
	Breeding region (Segovia)	169.34 ± 174.01	0.973	.331
	Breeding region (Cádiz)	950.40 ± 139.34	6.821	**<.001**
	Breeding region (Cazorla)	419.20 ± 84.79	4.944	**<.001**
	Breeding region (Pyrenees)	649.23 ± 108.33	5.993	**<.001**
Monthly home‐range fidelity	Season (spring)	0.439 ± 0.046	9.502	**<.001**
	Season (summer)	0.629 ± 0.046	13.549	**<.001**
	Season (autumn)	0.315 ± 0.047	6.653	**<.001**
	Sex (male)	0.267 ± 0.058	4.543	**<.001**
	Breeding region (Segovia)	0.691 ± 0.147	4.688	**<.001**
	Breeding region (Cádiz)	−0.099 ± 0.135	−0.729	.465
	Breeding region (Cazorla)	0.253 ± 0.096	2.635	**.008**
	Breeding region (Pyrenees)	0.255 ± 0.112	2.279	**.022**

*Note*: Season, Sex, and Breeding region variables were coded as a factor, being “Winter,” “Female,” and “Alto Ebro” the reference values for statistical comparisons. Significant values are highlighted in bold.

Abbreviation: SE, Standard error.

**FIGURE 3:**
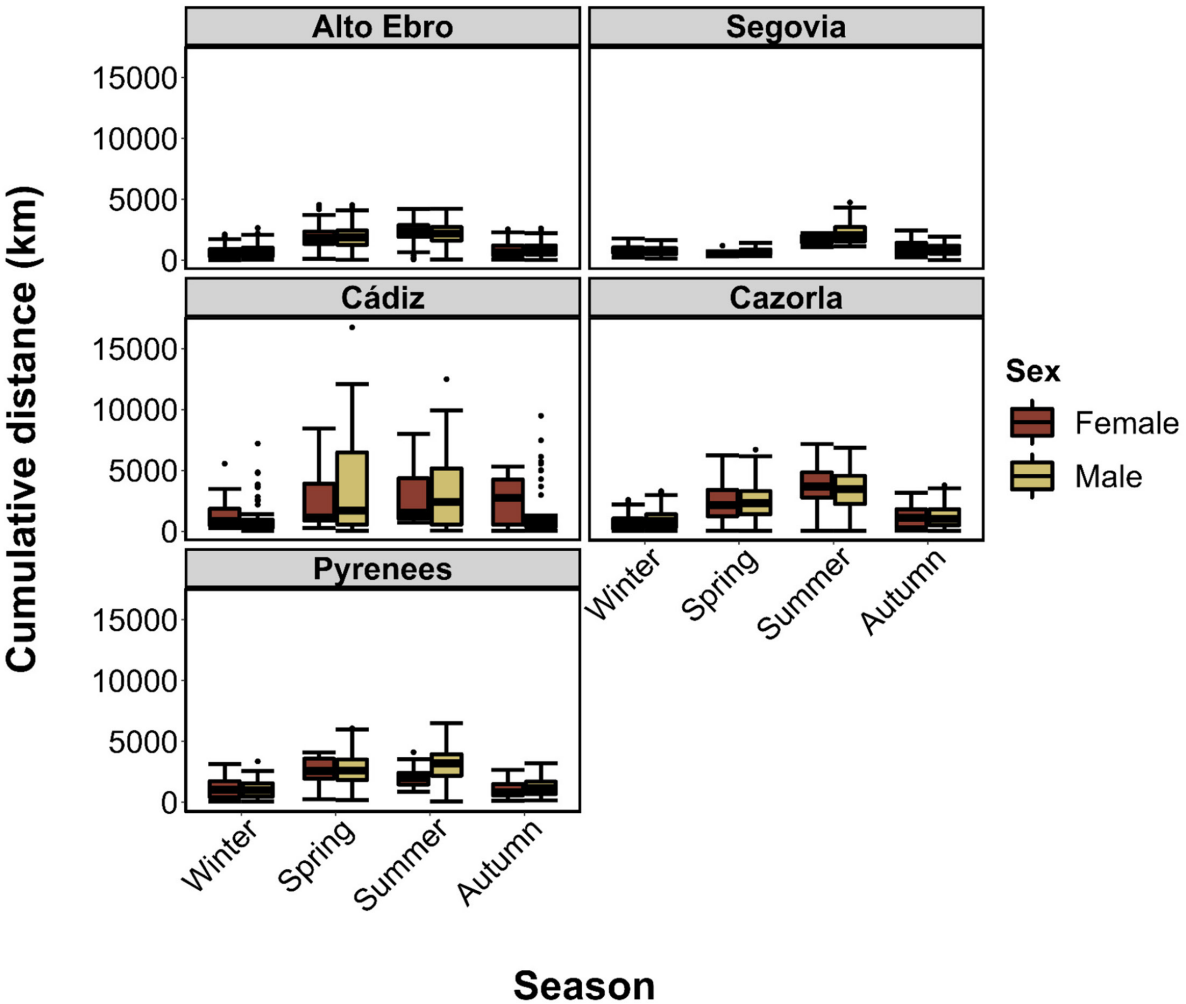
Mean monthly cumulative distance traveled (in km) of 127 adult Griffon Vultures (43 females and 84 males) tagged in northern (Alto Ebro and Pyrenees), central (Segovia), and southern (Cádiz and Cazorla) Spain. The standard deviation is shown as error bars.

Monthly cumulative distances traveled were similar for both females and males (Tables 2 and 3; Figure 3). However, birds traveled longer distances during spring and summer than during autumn and winter (Table S2). Individuals from Alto Ebro, Segovia, and Pyrenees traveled larger monthly distances than those of Cazorla and Cádiz (Table S2; Figure 3).

**FIGURE 4:**
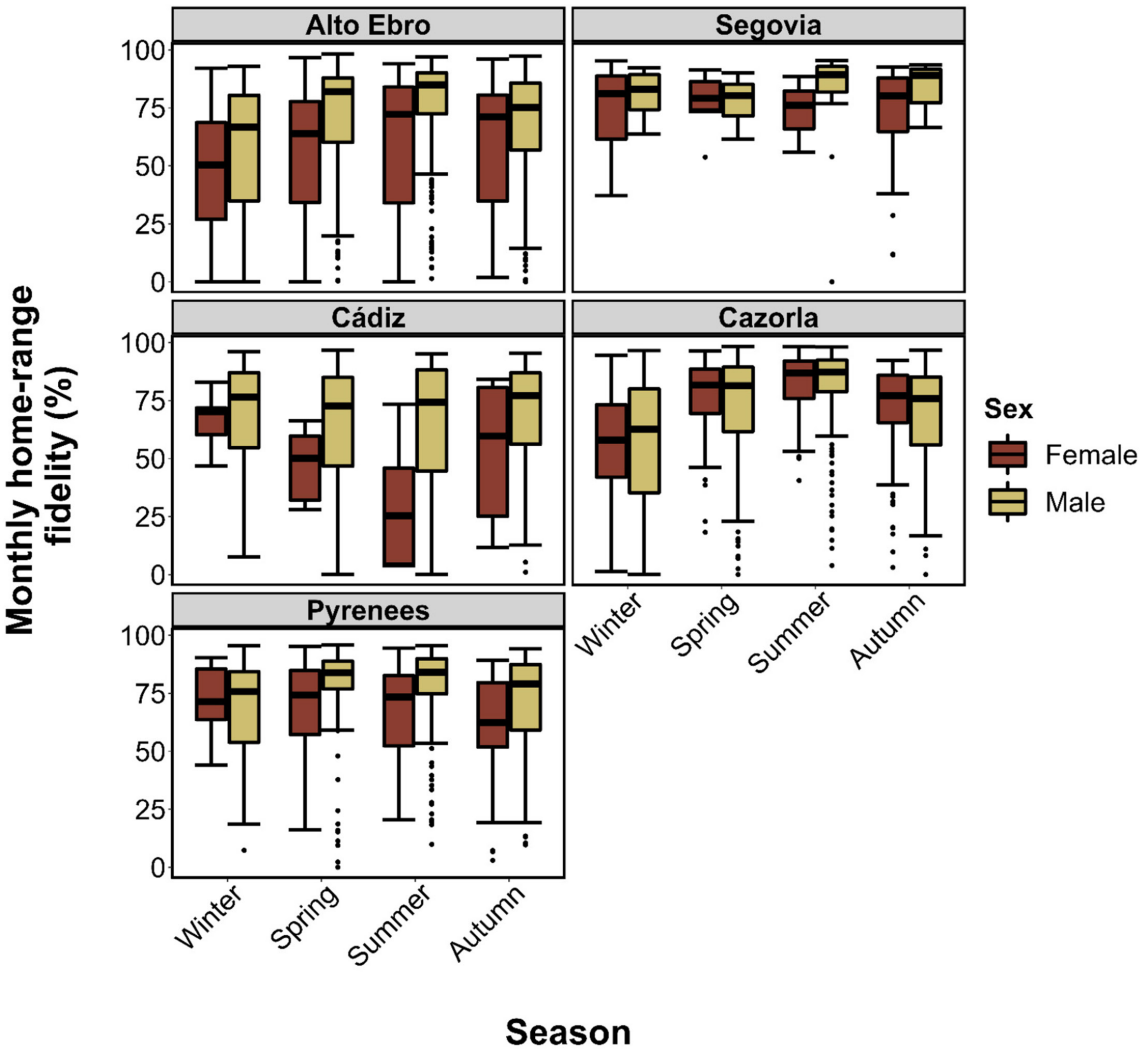
Differences in the monthly home‐range fidelity (in %) at an individual level between females and males in different seasons of 127 adult Griffon Vultures (43 females and 84 males) tagged in northern (Alto Ebro and Pyrenees), central (Segovia), and southern (Cádiz and Cazorla) Spain. The standard deviation is shown as error bars.

The monthly home‐range fidelity was slightly higher for males than females, and during spring and summer, being the lowest in autumn and winter. Likewise, there were differences in fidelity between breeding regions. Individuals from Central Spain (Segovia) showed higher fidelity over time than those from the north (Alto Ebro, Pyrenees) and southern Spain (Cádiz, Cazorla) (Tables 2 and 3 and Table S2; Figure 4).

## 4 DISCUSSION

Griffon Vulture movements varied between seasons, breeding regions and sexes. We found that movements were larger during spring and summer, which is similar to other soaring raptors such as Bearded Vulture or Bonelli's Eagle (see Margalida et al., 2016; Pérez‐García et al., 2013). This could be related to the food requirements associated with reproduction, which may force vultures to prospect larger areas, seeking for dispersed and unpredictable carcasses to satisfy the food requirements of the offspring and themselves (Carrete & Donázar, 2005). However, seasonal differences could be also explained by longer days (and therefore more time to forage) and better atmospheric conditions particularly during summer, minimizing energy expenditure during long‐range movements (see Martin‐Díaz et al., 2020). Similarly, differences in monthly home‐range size and cumulative distance traveled between regions could be explained by differences in both the importance of predictable and nonpredictable food availability and the ability to move due to better flight conditions due to the presence of thermal uplifts (Scacco et al., 2021). For instance, the southern populations may experience a higher thermal uplift availability (mainly due to warmer climatic conditions in summer), thus minimizing the energy expenditure while increasing the movement capacity of birds (e.g., see Scacco et al., 2019).

Differences in foraging performance between sexes are common in mammals and birds and are often due to differences in body size and parental duties (Lewis et al., 2002), In monomorphic species such as the Griffon Vulture, these differences might be associated with energetic and nutritional requirements for reproduction (Bennison et al., 2022; Pinet et al., 2012). Our results showed that females have larger home‐ranges and travel farther than males. These findings align with other studies in which the same dataset was analyzed and where females exhibited larger traveled distances than males during reproduction (see Delgado‐González et al., 2022; Gangoso et al., 2021), including other vulture species (see Bamford et al., 2007; García‐Jiménez et al., 2018; Kane et al., 2015; Krüger et al., 2014; Margalida et al., 2016).

Contrary to our expectations, we also found sex differences in the monthly home‐ranges fidelity. Males showed greater fidelity than females, indicating that the latter use different areas throughout the year. This gender variation in seasonal fidelity may respond to differences in resource selectivity (Delgado‐González et al., 2022; Hertel et al., 2020). In fact, according to Fernández‐Gómez et al. (2022) males may be more prone to feed on predictable resources such as supplementary feeding stations or vulture restaurants, while females may rely on more ephemeral and less clumped food resources. Thus, there may be parallel strategies in the large‐scale exploitation of space and, therefore, sexual spatial segregation (see also Perrig et al., 2021). Alternatively, the fact that males show a greater fidelity of their monthly home‐ranges throughout the year and that it is higher during the spring–summer period could be related to their greater territoriality. Males may be more involved in nest building and guarding (Xirouchakis & Mylonas, 2007), which might be a response to competence for nesting sites (see Zuberogoitia et al., 2018). Interestingly, females not only showed higher extension of home‐ranges than males but also exhibited lower site fidelity. All this reinforces the argument that females might forage more in different locations throughout the year, therefore, showing lesser home‐range fidelity over time than males. These sex and seasonal differences in individual home‐range fidelity were similar between breeding regions except in the case of Cádiz and Segovia, where females exhibited lower fidelity during spring/summer.

Differences (or lack of them) between breeding regions in individual movement patterns indicate that local effects not explored in this work may be affecting them. This is evidenced by the low variance explained by the fixed factors of the best models and the high variance explained by random factors (e.g., home‐range size fidelity models). The latter indicates that high interindividual differences in home‐range area and fidelity exist. Moreover, there are variables, such as distance to trophic resources, colony size, or habitat type, that perhaps could improve the results from our models and better explain breeding region level differences in the evaluated parameters (see Cecere et al., 2018; Delgado‐González et al., 2022; Harel et al., 2017). Finally, it should be taken into account that the effect of differences in the sampling duration of individuals of some breeding regions also affects the observed home‐range sizes which may have led to an underestimation of this and other parameters such as cumulative distance or home‐range fidelity.

## 5 CONCLUDING REMARKS

Our work adds evidence to the spatial requirements of large soaring birds such as vultures, and the importance of individual and regional differences in explaining their movement patterns. Contrary to our expectations, we found sex‐dependent spatial segregation in this monomorphic species, maybe related to behavioral differences between males and females, particularly during the breeding period. Moreover, we observed that vultures showed larger home‐ranges and cumulative distance traveled during the breeding period, when site‐fidelity is higher, with females traveling further. Finally, despite high levels of variation in home‐range sizes between breeding regions, there were no clear differences in site fidelity between breeding regions over time, which may indicate stability in the home‐range. Our findings open new promising avenues for research on interindividual differences in optimal foraging, and on the intrinsic and extrinsic factors operating at multiple levels (Williams & Safi, 2021). Beyond this, increasing our knowledge of how these patterns translate into regional differences (or lack thereof) is crucial in predicting population dynamics through movement ecology (Shaw, 2020).

Importantly, our results add information that is crucial may be decisive for the effective management and conservation of highly mobile species which require protective measures to be implemented at large spatial scales. For example, differences in the home‐range sizes between females and males may indicate different levels of exposure to well‐known threats to species (e.g., poisoning). This in fact could have direct effects on population dynamics in the long term, in particular, in those areas where overall individual survival is low (see Arrondo, Sanz‐Aguilar, et al., 2020). Additionally, the observed patterns of interpopulation and seasonal differences in movement ranges highlight the need to disentangle the risk factors for affecting different populations. In particular, in those places where installation of wind energy facilities is expected in the near future that may pose serious threat for the species (Pérez‐García et al., 2022; Serrano et al., 2020). Finally, the large movement patterns of this species highlight the need to implement transboundary conservation plans (see Lambertucci et al., 2014) and closely evaluate the sensitivity to different risk factors operating at each region to design a coordinated response of all countries involved in species conservation.

## AUTHOR CONTRIBUTIONS

**Jon Morant:** Conceptualization (equal); data curation (lead); formal analysis (lead); investigation (equal); methodology (lead). **Eneko Arrondo:** Supervision (supporting); validation (supporting); writing – original draft (supporting); writing – review and editing (supporting). **José Antonio Sánchez‐Zapata:** Supervision (supporting); validation (supporting); writing – original draft (supporting); writing – review and editing (supporting). **José Antonio Donázar:** Supervision (supporting); validation (supporting); writing – original draft (supporting); writing – review and editing (supporting). **Ainara Cortés‐Avizanda:** Supervision (supporting); validation (supporting); writing – original draft (supporting); writing – review and editing (supporting). **Manuel De La Riva:** Data curation (supporting); supervision (supporting); validation (supporting); writing – original draft (supporting); writing – review and editing (supporting). **Guillermo Blanco:** Supervision (supporting); validation (supporting); writing – original draft (supporting); writing – review and editing (supporting). **Félix Martínez:** Supervision (supporting); validation (supporting). **Juan Oltra:** Supervision (supporting); validation (supporting); writing – original draft (supporting); writing – review and editing (supporting). **Martina Carrete:** Supervision (supporting); validation (supporting); writing – original draft (supporting); writing – review and editing (supporting). **Antoni Margalida:** Supervision (supporting); validation (supporting); writing – original draft (supporting); writing – review and editing (supporting). **Pilar Oliva‐Vidal:** Supervision (supporting); validation (supporting); writing – original draft (supporting); writing – review and editing (supporting). **Jose María Martínez:** Supervision (supporting); validation (supporting). **David Serrano:** Supervision (supporting); validation (supporting); writing – original draft (supporting); writing – review and editing (supporting). **Juan Manuel Pérez‐García:** Conceptualization (equal); data curation (equal); formal analysis (equal); investigation (equal); methodology (equal); project administration (equal); supervision (equal); validation (equal); writing – original draft (equal); writing – review and editing (equal).

## CONFLICT OF INTEREST STATEMENT

The authors declare no competing interests.

## Supporting information


Table S1–S2

## Data Availability

The data and scripts used in the analyses are publicly available at Zenodo data repository (https://doi.org/10.5281/zenodo.7500857).

## References

[ece39817-bib-0001] Almaraz, P. , Martínez, F. , Morales‐Reyes, Z. , Sánchez‐Zapata, J. A. , & Blanco, G. (2022). Long‐term demographic dynamics of a keystone scavenger disrupted by human‐induced shifts in food availability. Ecological Applications, 32, e2579. 35279905 10.1002/eap.2579

[ece39817-bib-0002] Arkumarev, V. , Dobrev, D. , Stamenov, A. , Delchev, A. , & Stoychev, S. (2021). Seasonal and age‐specific dynamics of the griffon Vulture's home range and movements in the eastern Rhodopes. Ornis Hungarica, 29, 81–92.

[ece39817-bib-0003] Arrondo, E. , Moleón, M. , Cortés‐Avizanda, A. , Jiménez, J. , Beja, P. , Sánchez‐Zapata, J. A. , & Donázar, J. A. (2018). Invisible barriers: Differential sanitary regulations constrain vulture movements across country borders. Biological Conservation, 219, 46–52.

[ece39817-bib-0005] Arrondo, E. , Sanz‐Aguilar, A. , Pérez‐García, J. M. , Cortés‐Avizanda, A. , Sánchez‐Zapata, J. A. , & Donázar, J. A. (2020). Landscape anthropization shapes the survival of a top avian scavenger. Biodiversity and Conservation, 29, 1411–1425.

[ece39817-bib-0006] Bamford, A. J. , Diekmann, M. , Monadjem, A. , & Mendelsohn, J. (2007). Ranging behaviour of cape vultures Gyps coprotheres from an endangered population in Namibia. Bird Conservation International, 17, 331–339.

[ece39817-bib-0007] Bennison, A. , Giménez, J. , Quinn, J. L. , Green, J. A. , & Jessopp, M. (2022). A bioenergetics approach to understanding sex differences in the foraging behaviour of a sexually monomorphic species. Royal Society Open Science, 9, 210520. 35116139 10.1098/rsos.210520 PMC8790366

[ece39817-bib-0010] Bodey, T. W. , Cleasby, I. R. , Bell, F. , Parr, N. , Schultz, A. , Votier, S. C. , & Bearhop, S. (2018). A phylogenetically controlled meta‐analysis of biologging device effects on birds: Deleterious effects and a call for more standardized reporting of study data. Methods in Ecology and Evolution, 9, 946–955.

[ece39817-bib-0011] Bolnick, D. I. , Svanbäck, R. , Fordyce, J. A. , Yang, L. H. , Davis, J. M. , Hulsey, C. D. , & Forister, M. L. (2003). The ecology of individuals: Incidence and implications of individual specialization. American Naturalist, 161, 1–28. 10.1086/343878 12650459

[ece39817-bib-0012] Brooks, M. E. , Kristensen, K. , Van Benthem, K. J. , Magnusson, A. , Berg, C. W. , Nielsen, A. , & Bolker, B. M. (2017). glmmTMB balances speed and flexibility among packages for zero‐inflated generalized linear mixed modeling. The R Journal, 9, 378–400.

[ece39817-bib-0013] Burnham, K. P. , & Anderson, D. R. (2002). Model selection and multimodel inference: A practical information‐theoretic approach (2ª). Ecological Modelling. Springer Science & Business Media.

[ece39817-bib-0014] Carrete, M. , & Donázar, J. A. (2005). Application of central‐place foraging theory shows the importance of Mediterranean dehesas for the conservation of the cinereous vulture, Aegypius monachus. Biological Conservation, 126, 582–590.

[ece39817-bib-0015] Cecere, J. G. , Bondì, S. , Podofillini, S. , Imperio, S. , Griggio, M. , Fulco, E. , Curcio, A. , Ménard, D. , Mellone, U. , Saino, N. , Serra, L. , Sarà, M. , & Rubolini, D. (2018). Spatial segregation of home ranges between neighbouring colonies in a diurnal raptor. Scientific Reports, 8, 1–9. 30082763 10.1038/s41598-018-29933-2 PMC6078973

[ece39817-bib-0016] Cortés‐Avizanda, A. , Carrete, M. , & Donázar, J. A. (2010). Managing supplementary feeding for avian scavengers: Guidelines for optimal design using ecological criteria. Biological Conservation, 143, 1707–1715. 10.1016/j.biocon.2010.04.016

[ece39817-bib-0017] Cortés‐Avizanda, A. , Jovani, R. , Carrete, M. , & Donázar, J. A. (2012). Resource unpredictability promotes species diversity and coexistence in an avian scavenger guild: A field experiment. Ecology, 93, 2570–2579. 23431588 10.1890/12-0221.1

[ece39817-bib-0018] Cortés‐Avizanda, A. , Jovani, R. , Donázar, J. A. , & Grimm, V. (2014). Bird sky networks: How do avian scavengers use social information to find carrion? Ecology, 95, 1799–1808. 25163114 10.1890/13-0574.1

[ece39817-bib-0019] Costa‐Pereira, R. , Moll, R. J. , Jesmer, B. R. , & Jetz, W. (2022). Animal tracking moves community ecology: Opportunities and challenges. Journal of Animal Ecology, 91, 1334–1344. 35388473 10.1111/1365-2656.13698 PMC10286655

[ece39817-bib-0020] Del Moral, J. C. , & Molina, B. (2018). El buitre leonado en España, población reproductora en 2018 y método de censo. SEO/BirdLife.

[ece39817-bib-0021] Delgado‐González, A. , Cortés‐Avizanda, A. , Serrano, D. , Arrondo, E. , Duriez, O. , Margalida, A. , Carrete, M. , Oliva‐Vidal, P. , Sourp, E. , Morales‐Reyes, Z. , García‐Barón, I. , de la Riva, M. , Sánchez‐Zapata, J. A. , & Donázar, J. A. (2022). Apex scavengers from different European populations converge at threatened savannah landscapes. Scientific Reports, 12, 1–10. 35169202 10.1038/s41598-022-06436-9 PMC8847400

[ece39817-bib-0022] Donázar, J. A. (1993). Los buitres ibéricos: Biología y conservación. JM Reyero.

[ece39817-bib-0023] Edelhoff, H. , Signer, J. , & Balkenhol, N. (2016). Path segmentation for beginners: An overview of current methods for detecting changes in animal movement patterns. Movement Ecology, 4, 1–21. 27595001 10.1186/s40462-016-0086-5 PMC5010771

[ece39817-bib-0024] Fernández‐Gómez, L. , Cortes‐Avizanda, A. , Arrondo, E. , García‐Alfonso, M. , Ceballos, O. , Montelio, E. , & Donazar, J. A. (2022). Vultures feeding on the dark side: Current sanitary regulations may not be enough. Bird Conservation International, 32, 1–19.

[ece39817-bib-0025] Fieberg, J. , & Kochanny, C. O. (2005). Quantifying home‐range overlap: The importance of the utilization distribution. The Journal of Wildlife Management, 69, 1346–1359.

[ece39817-bib-0026] Fluhr, J. , Benhamou, S. , Peyrusque, D. , & Duriez, O. (2021). Space use and time budget in two populations of griffon vultures in contrasting landscapes. Journal of Raptor Research, 55, 425–437.

[ece39817-bib-0027] Fridolfsson, A.‐K. , & Ellegren, H. (1999). A simple and universal method for molecular sexing of non‐ratite birds. Journal of Avian Biology, 34, 116–121.

[ece39817-bib-0028] Gangoso, L. , Cortés‐Avizanda, A. , Sergiel, A. , Pudifoot, B. , Miranda, F. , Muñoz, J. , Delgado‐Gonzázlez, A. , Moleón, M. , Sánchez‐Zapata, J. A. , Arrondo, E. , & Donázar, J. A. (2021). Avian scavengers living in anthropized landscapes have shorter telomeres and higher levels of glucocorticoid hormones. Science of the Total Environment, 782, 146920.

[ece39817-bib-0029] García‐Jiménez, R. , Pérez‐García, J. M. , & Margalida, A. (2018). Drivers of daily movement patterns affecting an endangered vulture flight activity. BMC Ecology, 18, 1–15. 30268111 10.1186/s12898-018-0195-7 PMC6162909

[ece39817-bib-0030] García‐Ripollés, C. , López‐López, P. , & Urios, V. (2011). Ranging behaviour of non‐breeding Eurasian griffon vultures *Gyps fulvus*: A GPS‐telemetry study. Acta Ornithologica, 46, 127–134.

[ece39817-bib-0076] Goode, K. , & Rey, K. (2019). ggResidpanel: Panels and Interactive Versions of Diagnostic Plots using ‘ggplot2’ R package version 0.3.0. https://CRAN.R‐project.org/package=ggResidpanel

[ece39817-bib-0031] Hansson, L. A. , Akesson, S. , & Åkesson, S. (2014). Animal movement across scales. Oxford University Press.

[ece39817-bib-0032] Harel, R. , Spiegel, O. , Getz, W. M. , & Nathan, R. (2017). Social foraging and individual consistency in following behaviour: Testing the information Centre hypothesis in free‐ranging vultures. Proceedings of the Royal Society B: Biological Sciences, 284, 20162654. 10.1098/rspb.2016.2654 PMC5394657 28404771

[ece39817-bib-0033] Hertel, A. G. , Niemelä, P. T. , Dingemanse, N. J. , & Mueller, T. (2020). A guide for studying among‐individual behavioral variation from movement data in the wild. Movement Ecology, 8, 1–18. 32612837 10.1186/s40462-020-00216-8 PMC7325061

[ece39817-bib-0034] Jobson, B. , Wolter, K. , Jordan, L. , Monadjem, A. , & Rowcliffe, J. M. (2021). Home range and habitat selection of captive‐bred and rehabilitated cape vultures Gyps coprotheres in southern Africa. Oryx, 55, 607–612.

[ece39817-bib-0035] Kane, A. , Jackson, A. L. , Monadjem, A. , Colomer, M. A. , & Margalida, A. (2015). Carrion ecology modelling for vulture conservation: Are vulture restaurants needed to sustain the densest breeding population of the African white‐backed vulture? Animal Conservation, 18, 279–286. 10.1111/acv.12169

[ece39817-bib-0036] Katzner, T. E. , & Arlettaz, R. (2020). Evaluating contributions of recent tracking‐based animal movement ecology to conservation management. Frontiers in Ecology and Evolution, 7, 519.

[ece39817-bib-0037] Kie, J. G. , Matthiopoulos, J. , Fieberg, J. , Powell, R. A. , Cagnacci, F. , Mitchell, M. S. , Gaillard, J. M. , & Moorcroft, P. R. (2010). The home‐range concept: Are traditional estimators still relevant with modern telemetry technology? Philosophical Transactions of the Royal Society B: Biological Sciences, 365, 2221–2231. 10.1098/rstb.2010.0093 PMC2894967 20566499

[ece39817-bib-0039] Krüger, S. , Reid, T. , & Amar, A. (2014). Differential range use between age classes of southern African bearded vultures Gypaetus barbatus. PLoS One, 9, e114920. 25551614 10.1371/journal.pone.0114920 PMC4281122

[ece39817-bib-0040] Lambertucci, S. A. , Alarcón, P. A. , Hiraldo, F. , Sanchez‐Zapata, J. A. , Blanco, G. , & Donázar, J. A. (2014). Apex scavenger movements call for transboundary conservation policies. Biological Conservation, 170, 145–150.

[ece39817-bib-0041] Lenth, R. V. (2022). Emmeans: Estimated marginal means, aka least‐squares means. R package versión 1.7.4‐1. https://CRAN.R‐project.org/package=emmeans

[ece39817-bib-0042] Lewis, S. , Benvenuti, S. , Dall'Antonia, L. , Griffiths, R. , Money, L. , Sherratt, T. N. , Wanless, S. , & Hamer, K. C. (2002). Sex‐specific foraging behaviour in a monomorphic seabird. Proceedings of the Royal Society of London B: Biological Sciences, 269, 1687–1693. 10.1098/rspb.2002.2083 PMC1691079 12204129

[ece39817-bib-0043] Lüdecke, D. , Ben‐Shachar, M. S. , Patil, I. , Waggoner, P. , & Makowski, D. (2021). Performance: An R package for assessment, comparison and testing of statistical models. Journal of Open Source Software, 6, 3139. 10.21105/joss.03139

[ece39817-bib-0044] Margalida, A. , Oliva‐Vidal, P. , Llamas, A. , & Colomer, M. À. (2018). Bioinspired models for assessing the importance of transhumance and transboundary management in the conservation of European avian scavengers. Biological Conservation, 228, 321–330.

[ece39817-bib-0045] Margalida, A. , Pérez‐García, J. M. , Afonso, I. , & Moreno‐Opo, R. (2016). Spatial and temporal movements in Pyrenean bearded vultures (Gypaetus barbatus): Integrating movement ecology into conservation practice. Scientific Reports, 6, 1–12. 27779179 10.1038/srep35746 PMC5078842

[ece39817-bib-0046] Martin‐Díaz, P. , Cortés‐Avizanda, A. , Serrano, D. , Arrondo, E. , Sánchez‐Zapata, J. A. , & Donázar, J. A. (2020). Rewilding processes shape the use of Mediterranean landscapes by an avian top scavenger. Scientific Reports, 10, 1–12. 32071326 10.1038/s41598-020-59591-2 PMC7028937

[ece39817-bib-0047] Monsarrat, S. , Benhamou, S. , Sarrazin, F. , Bessa‐Gomes, C. , Bouten, W. , & Duriez, O. (2013). How predictability of feeding patches affects home range and foraging habitat selection in avian social scavengers? PLoS One, 8, e53077. 23301024 10.1371/journal.pone.0053077 PMC3536817

[ece39817-bib-0048] Morant, J. , Arrondo, E. , Cortés‐Avizanda, A. , Moleón, M. , Donázar, J. A. , Sánchez‐Zapata, J. A. , López‐López, P. , Ruiz‐Villar, H. , Zuberogoitia, I. , Morales‐Reyes, Z. , Naves‐Alegre, L. , & Sebastián‐González, E. (2022). Large‐scale quantification and correlates of ungulate carrion production in the Anthropocene. Ecosystems, 1–14. 10.1007/s10021-022-00763-8

[ece39817-bib-0049] Nathan, R. , Getz, W. M. , Revilla, E. , Holyoak, M. , Kadmon, R. , Saltz, D. , & Smouse, P. E. (2008). A movement ecology paradigm for unifying organismal movement research. Proceedings of the National Academy of Sciences of the United States of America, 105, 19052–19059. 19060196 10.1073/pnas.0800375105 PMC2614714

[ece39817-bib-0050] Nathan, R. , Spiegel, O. , Fortmann‐Roe, S. , Harel, R. , Wikelski, M. , & Getz, W. M. (2012). Using tri‐axial acceleration data to identify behavioral modes of free‐ranging animals: General concepts and tools illustrated for griffon vultures. Journal of Experimental Biology, 215, 986–996. 22357592 10.1242/jeb.058602 PMC3284320

[ece39817-bib-0051] Pérez‐García, J. M. , Margalida, A. , Afonso, I. , Ferreiro, E. , Gardiazábal, A. , Botella, F. , & Sánchez‐Zapata, J. A. (2013). Interannual home range variation, territoriality and overlap in breeding Bonelli's eagles (Aquila fasciata) tracked by GPS satellite telemetry. Journal of Ornithology, 154, 63–71.

[ece39817-bib-0052] Pérez‐García, J. M. , Morant, J. , Arrondo, E. , Sebastián‐González, E. , Lambertucci, S. A. , Santangeli, A. , Margalida, A. , Sánchez‐Zapata, J. A. , Blanco, G. , Donázar, J. A. , Carrete, M. , & Serrano, D. (2022). Priority areas for conservation alone are not a good proxy for predicting the impact of renewable energy expansion. Proceedings of the National Academy of Sciences of the United States of America, 119, e2204505119. 35878057 10.1073/pnas.2204505119 PMC9388154

[ece39817-bib-0053] Perrig, P. L. , Lambertucci, S. A. , Alarcón, P. A. , Middleton, A. D. , Padró, J. , Plaza, P. I. , Blanco, G. , Sánchez‐Zapata, J. A. , Donázar, J. A. , & Pauli, J. N. (2021). Limited sexual segregation in a dimorphic avian scavenger, the Andean condor. Oecologia, 196, 77–88. 33837824 10.1007/s00442-021-04909-8

[ece39817-bib-0055] Pinet, P. , Jaquemet, S. , Phillips, R. A. , & Le Corre, M. (2012). Sex‐specific foraging strategies throughout the breeding season in a tropical, sexually monomorphic small petrel. Animal Behaviour, 83, 979–989. 10.1016/j.anbehav.2012.01.019

[ece39817-bib-0056] R Core Team . (2021). R: A language and environment for statistical computing. R Foundation for Statistical Computing. https://www.R‐project.org/

[ece39817-bib-0057] Scacco, M. , Arrondo, E. , Donázar, J. A. , Flack, A. , Sánchez‐Zapata, J. A. , Duriez, O. , Wikelski, M. , & Safi, K. (2021). The species‐specificity of energy landscapes for soaring birds, and its consequences for transferring suitability models across species. Landscape Ecology, 38, 239–252. 10.1007/s10980-022-01551-4

[ece39817-bib-0058] Scacco, M. , Flack, A. , Duriez, O. , Wikelski, M. , & Safi, K. (2019). Static landscape features predict uplift locations for soaring birds across Europe. Royal Society Open Science, 6, 181440. 30800386 10.1098/rsos.181440 PMC6366234

[ece39817-bib-0059] Serrano, D. , Margalida, A. , Pérez‐García, J. M. , Juste, J. , Traba, J. , Valera, F. , Carrete, M. , Aihartza, A. , Real, J. , Mañosa, S. , Flaquer, C. , Garin, I. , Morales, M. B. , Tomás Alcalde, J. , Arroyo, B. , Sánchez‐Zapata, J. A. , Blanco, G. , Negro, J. J. , Tella, J. L. , … Donázar, J. A. (2020). Renewables in Spain threaten biodiversity. Science, 370, 1282–1283. 33303607 10.1126/science.abf6509

[ece39817-bib-0060] Shaffer, S. A. , Cockerham, S. , Warzybok, P. , Bradley, R. W. , Jahncke, J. , Clatterbuck, C. A. , Lucia, M. , Jelincic, J. A. , Cassell, A. L. , Kelsey, E. C. , & Adams, J. (2017). Population‐level plasticity in foraging behavior of western gulls (*Larus occidentalis*). Movement Ecology, 5, 1–13. 29270295 10.1186/s40462-017-0118-9 PMC5735870

[ece39817-bib-0061] Shaw, A. K. (2020). Causes and consequences of individual variation in animal movement. Movement Ecology, 8, 1–12. 32099656 10.1186/s40462-020-0197-x PMC7027015

[ece39817-bib-0062] Signer, J. , Fieberg, J. , & Avgar, T. (2019). Animal movement tools (amt): R package for managing tracking data and conducting habitat selection analyses. Ecology and Evolution, 9, 880–890. 30766677 10.1002/ece3.4823 PMC6362447

[ece39817-bib-0063] Silva, I. , Fleming, C. H. , Noonan, M. J. , Alston, J. , Folta, C. , Fagan, W. F. , & Calabrese, J. M. (2022). Autocorrelation‐informed home range estimation: A review and practical guide. Methods in Ecology and Evolution, 13, 534–544.

[ece39817-bib-0064] Spiegel, O. , Harel, R. , Centeno‐Cuadros, A. , Hatzofe, O. , Getz, W. M. , & Nathan, R. (2015). Moving beyond curve fitting: Using complementary data to assess alternative explanations for long movements of three vulture species. The American Naturalist, 185, E44–E54.

[ece39817-bib-0065] Spiegel, O. , Harel, R. , Getz, W. M. , & Nathan, R. (2013). Mixed strategies of griffon vultures' (Gyps fulvus) response to food deprivation lead to a hump‐shaped movement pattern. Movement Ecology, 1, 1–12. 25709819 10.1186/2051-3933-1-5 PMC4337378

[ece39817-bib-0067] Thaker, M. , Gupte, P. R. , Prins, H. H. , Slotow, R. , & Vanak, A. T. (2019). Fine‐scale tracking of ambient temperature and movement reveals shuttling behavior of elephants to water. Frontiers in Ecology and Evolution, 7, 4.

[ece39817-bib-0068] Tucker, M. A. , Böhning‐Gaese, K. , Fagan, W. F. , Fryxell, J. M. , Van Moorter, B. , Alberts, S. C. , Ali, A. H. , Allen, A. M. , Attias, N. , Avgar, T. , Bartlam‐Brooks, H. , Bayarbaatar, B. , Belant, J. L. , Bertassoni, A. , Beyer, D. , Bidner, L. , van Beest, F. M. , Blake, S. , Blaum, N. , … Mueller, T. (2018). Moving in the Anthropocene: Global reductions in terrestrial mammalian movements. Science, 359, 466–469. 29371471 10.1126/science.aam9712

[ece39817-bib-0070] Williams, H. J. , & Safi, K. (2021). Certainty and integration of options in animal movement. Trends in Ecology & Evolution, 36, 990–999. 34303526 10.1016/j.tree.2021.06.013

[ece39817-bib-0071] Winner, K. , Noonan, M. J. , Fleming, C. H. , Olson, K. A. , Mueller, T. , Sheldon, D. , & Calabrese, J. M. (2018). Statistical inference for home range overlap. Methods in Ecology and Evolution, 9, 1679–1691. 10.1111/2041-210X.13027

[ece39817-bib-0072] Xirouchakis, S. , Grivas, C. , Andreou, G. , & Georgopoulou, E. (2021). Home range size, space use and resource selection of griffon vultures in an insular environment. Journal of Zoology, 314, 116–131. 10.1111/jzo.12868

[ece39817-bib-0073] Xirouchakis, S. , & Mylonas, M. (2007). Breeding behaviour and parental care in the griffon vulture Gyps fulvus on the Island of Crete (Greece). Ethology Ecology & Evolution, 19, 1–26.

[ece39817-bib-0074] Zuberogoitia, I. , De La Puente, J. , Elorriaga, J. , Alonso, R. , Palomares, L. E. , & Martínez, J. E. (2013). The flight feather molt of griffon vultures (Gyps fulvus) and associated biological consequences. Journal of Raptor Research, 47, 292–303.

[ece39817-bib-0075] Zuberogoitia, I. , Martínez, J. E. , González‐Oreja, J. A. , Pérez de Ana, J. M. , & Zabala, J. (2018). Factors affecting population regulation of a colonial vulture. Ibis, 161, 878–889.

