# Supplementary material for: Large‐scale movement patterns in a social vulture are influenced by seasonality, sex, and breeding region
Source: Ecol Evol. 2023 Feb 8;13(2):e9817. doi: 10.1002/ece3.9817 (PMC9909000; doi:10.1002/ece3.9817)
Supplement: Supplementary file 1 — Table S1–S2 [file ECE3-13-e9817-s001.docx]

**Supplementary material**

**Large-scale movement patterns in a social vulture are influenced by seasonality, sex and breeding region**

JON MORANT^1*^, ENEKO ARRONDO^1^, JOSÉ ANTONIO SÁNCHEZ-ZAPATA^1^, JOSÉ ANTONIO DONÁZAR^2^, AINARA CORTÉS-AVIZANDA^3^, MANUEL DE LA RIVA^2^, GUILLERMO BLANCO^4^, FÉLIX MARTÍNEZ^9^, JUAN OLTRA^4^, MARTINA CARRETE^4^, ANTONI MARGALIDA^5,6^, PILAR OLIVA-VIDAL^7^, JOSÉ MARIA MARTÍNEZ^8^, DAVID SERRANO^2^ & JUAN MANUEL PÉREZ-GARCÍA^1^

*Corresponding author: JON MORANT

Email: [jmorantetxebarria@gmail.com](mailto:jmorantetxebarria@gmail.com)

Twitter: @MorantJon

**Table S1.** Details of the GPS-tagged adult Griffon vultures (n=127) in Spain from 2014 to 2021.

| **animal-id** | **deploy-on-date** | **deploy-off-date** | **days** | **sex** | **population** | **Region** | **GPS** |
| --- | --- | --- | --- | --- | --- | --- | --- |
| T1L | 21/12/2015 | 23/11/2021 | 2164 | male | Alto Ebro | Navarra | E-Obs |
| T1N | 21/12/2015 | 08/07/2018 | 930 | female | Alto Ebro | Navarra | E-Obs |
| T1R | 21/12/2015 | 27/01/2019 | 1133 | male | Alto Ebro | Navarra | E-Obs |
| T1U | 21/12/2015 | 19/01/2017 | 395 | female | Alto Ebro | Navarra | E-Obs |
| T1W | 21/12/2015 | 10/08/2017 | 598 | male | Alto Ebro | Navarra | E-Obs |
| T1X | 21/12/2015 | 04/02/2021 | 1872 | female | Alto Ebro | Navarra | E-Obs |
| T20 | 21/12/2015 | 29/12/2015 | 8 | male | Alto Ebro | Navarra | E-Obs |
| T21 | 21/12/2015 | 09/05/2021 | 1966 | male | Alto Ebro | Navarra | E-Obs |
| T22 | 21/12/2015 | 17/09/2016 | 271 | male | Alto Ebro | Navarra | E-Obs |
| T24 | 21/12/2015 | 16/03/2020 | 1547 | female | Alto Ebro | Navarra | E-Obs |
| T25 | 21/12/2015 | 10/04/2022 | 2302 | male | Alto Ebro | Navarra | E-Obs |
| T2A | 17/01/2016 | 23/05/2016 | 127 | male | Alto Ebro | Navarra | E-Obs |
| T2C | 17/01/2016 | 11/08/2016 | 207 | male | Alto Ebro | Navarra | E-Obs |
| T2F | 17/01/2016 | 27/06/2021 | 1988 | female | Alto Ebro | Navarra | E-Obs |
| T2H | 17/01/2016 | 10/04/2022 | 2275 | male | Alto Ebro | Navarra | E-Obs |
| T2J | 17/01/2016 | 21/06/2018 | 886 | male | Alto Ebro | Navarra | E-Obs |
| T2L | 17/01/2016 | 13/06/2020 | 1609 | female | Alto Ebro | Navarra | E-Obs |
| T2M | 17/01/2016 | 01/08/2021 | 2023 | male | Alto Ebro | Navarra | E-Obs |
| T2N | 17/01/2016 | 02/08/2016 | 198 | male | Alto Ebro | Navarra | E-Obs |
| T2R | 17/01/2016 | 27/11/2018 | 1045 | female | Alto Ebro | Navarra | E-Obs |
| T2T | 17/01/2016 | 10/03/2016 | 53 | female | Alto Ebro | Navarra | E-Obs |
| T2U | 17/01/2016 | 09/04/2022 | 2274 | male | Alto Ebro | Navarra | E-Obs |
| T2V | 17/01/2016 | 10/04/2022 | 2275 | female | Alto Ebro | Navarra | E-Obs |
| T2W | 19/01/2016 | 29/08/2019 | 1318 | female | Alto Ebro | Navarra | E-Obs |
| T2X | 19/01/2016 | 01/06/2018 | 864 | female | Alto Ebro | Navarra | E-Obs |
| T30 | 19/01/2016 | 17/08/2019 | 1306 | female | Alto Ebro | Navarra | E-Obs |
| T31 | 19/01/2016 | 10/04/2020 | 1543 | female | Alto Ebro | Navarra | E-Obs |
| T33 | 19/01/2016 | 19/04/2019 | 1186 | female | Alto Ebro | Navarra | E-Obs |
| T35 | 19/01/2016 | 17/09/2016 | 242 | female | Alto Ebro | Navarra | E-Obs |
| T36 | 19/01/2016 | 15/11/2021 | 2127 | male | Alto Ebro | Navarra | E-Obs |
| T3T | 27/04/2016 | 20/11/2016 | 207 | female | Alto Ebro | Navarra | E-Obs |
| VWC | 24/03/2017 | 06/05/2017 | 43 | male | Alto Ebro | Navarra | E-Obs |
| VWJ | 24/03/2017 | 03/11/2018 | 589 | male | Alto Ebro | Navarra | E-Obs |
| VWT | 25/03/2017 | 07/11/2017 | 227 | male | Alto Ebro | Navarra | E-Obs |
| VWU | 25/03/2017 | 27/05/2017 | 63 | male | Alto Ebro | Navarra | E-Obs |
| VWX | 25/03/2017 | 08/05/2018 | 409 | female | Alto Ebro | Navarra | E-Obs |
| Japi PXF | 07/06/2019 | 10/04/2022 | 1039 | female | Alto Ebro | Euskadi | Ornitela |
| Belar U71 | 07/06/2019 | 10/04/2022 | 1039 | male | Alto Ebro | Euskadi | Ornitela |
| Oñati | 07/06/2019 | 10/04/2022 | 1039 | male | Alto Ebro | Euskadi | Ornitela |
| Pipi WJ9 | 07/06/2019 | 19/11/2019 | 166 | male | Alto Ebro | Euskadi | Ornitela |
| Urbia | 07/06/2019 | 10/04/2022 | 1039 | male | Alto Ebro | Euskadi | Ornitela |
| Aloña | 07/06/2019 | 28/04/2020 | 326 | male | Alto Ebro | Euskadi | Ornitela |
| Kirikixo | 07/06/2019 | 10/04/2022 | 1039 | male | Alto Ebro | Euskadi | Ornitela |
| Ur | 07/06/2019 | 10/04/2022 | 1038 | male | Alto Ebro | Euskadi | Ornitela |
| Amorrai | 07/06/2019 | 10/04/2022 | 1039 | male | Alto Ebro | Euskadi | Ornitela |
| Haize | 07/06/2019 | 10/04/2022 | 1038 | male | Alto Ebro | Euskadi | Ornitela |
| Txingurri | 07/06/2019 | 10/04/2022 | 1039 | male | Alto Ebro | Euskadi | Ornitela |
| Harri | 07/06/2019 | 10/04/2022 | 1038 | male | Alto Ebro | Euskadi | Ornitela |
| VUS01 | 25/05/2018 | 16/11/2020 | 906 | male | Cádiz | Andalucia | Ecotone |
| VUS02 | 03/08/2018 | 13/09/2021 | 1137 | male | Cádiz | Andalucia | Ecotone |
| VUS03 | 18/07/2018 | 18/09/2019 | 427 | male | Cádiz | Andalucia | Ecotone |
| VUS04 | 25/05/2018 | 19/08/2021 | 1182 | male | Cádiz | Andalucia | Ecotone |
| VUS05 | 09/07/2018 | 19/06/2020 | 711 | female | Cádiz | Andalucia | Ecotone |
| VUS06 | 15/07/2018 | 20/07/2019 | 370 | male | Cádiz | Andalucia | Ecotone |
| VUS07 | 15/07/2018 | 19/07/2019 | 369 | female | Cádiz | Andalucia | Ecotone |
| VUS08 | 12/07/2018 | 18/04/2022 | 1376 | male | Cádiz | Andalucia | Ecotone |
| VUS09 | 05/06/2018 | 19/08/2019 | 441 | male | Cádiz | Andalucia | Ecotone |
| VUS10 | 15/07/2018 | 17/11/2020 | 856 | male | Cádiz | Andalucia | Ecotone |
| VUS11 | 15/07/2018 | 19/07/2020 | 735 | male | Cádiz | Andalucia | Ecotone |
| VUS12 | 09/07/2018 | 16/12/2019 | 525 | male | Cádiz | Andalucia | Ecotone |
| L73 | 19/12/2014 | 08/04/2022 | 2667 | male | Cazorla | Andalucia | E-Obs |
| L8J | 17/12/2014 | 09/11/2021 | 2519 | male | Cazorla | Andalucia | E-Obs |
| T00 | 04/12/2014 | 02/04/2022 | 2676 | female | Cazorla | Andalucia | E-Obs |
| T01 | 04/12/2014 | 21/03/2022 | 2664 | female | Cazorla | Andalucia | E-Obs |
| T02 | 17/12/2014 | 11/10/2016 | 664 | male | Cazorla | Andalucia | E-Obs |
| T03 | 17/12/2014 | 29/04/2018 | 1229 | male | Cazorla | Andalucia | E-Obs |
| T05 | 17/12/2014 | 11/05/2021 | 2337 | male | Cazorla | Andalucia | E-Obs |
| T06 | 17/12/2014 | 21/08/2015 | 247 | male | Cazorla | Andalucia | E-Obs |
| T07 | 17/12/2014 | 25/02/2016 | 435 | male | Cazorla | Andalucia | E-Obs |
| T08 | 17/12/2014 | 08/04/2022 | 2669 | female | Cazorla | Andalucia | E-Obs |
| T09 | 17/12/2014 | 09/07/2021 | 2396 | male | Cazorla | Andalucia | E-Obs |
| T0A | 17/12/2014 | 09/03/2021 | 2274 | male | Cazorla | Andalucia | E-Obs |
| T0C | 17/12/2014 | 26/11/2021 | 2536 | male | Cazorla | Andalucia | E-Obs |
| T0H | 17/12/2014 | 25/04/2020 | 1956 | male | Cazorla | Andalucia | E-Obs |
| T0J | 17/12/2014 | 10/04/2022 | 2671 | male | Cazorla | Andalucia | E-Obs |
| T0L | 17/12/2014 | 13/09/2015 | 270 | male | Cazorla | Andalucia | E-Obs |
| T0U | 19/12/2014 | 03/02/2017 | 777 | female | Cazorla | Andalucia | E-Obs |
| T0V | 19/12/2014 | 18/12/2021 | 2556 | male | Cazorla | Andalucia | E-Obs |
| T0W | 19/12/2014 | 03/02/2016 | 411 | female | Cazorla | Andalucia | E-Obs |
| T0X | 19/12/2014 | 05/10/2016 | 656 | male | Cazorla | Andalucia | E-Obs |
| T10 | 19/12/2014 | 26/11/2015 | 342 | female | Cazorla | Andalucia | E-Obs |
| T11 | 19/12/2014 | 10/10/2021 | 2487 | male | Cazorla | Andalucia | E-Obs |
| T12 | 19/12/2014 | 12/09/2017 | 998 | male | Cazorla | Andalucia | E-Obs |
| T14 | 19/12/2014 | 12/11/2021 | 2520 | male | Cazorla | Andalucia | E-Obs |
| T15 | 19/12/2014 | 07/04/2022 | 2666 | female | Cazorla | Andalucia | E-Obs |
| T16 | 19/12/2014 | 18/09/2017 | 1004 | male | Cazorla | Andalucia | E-Obs |
| T17 | 19/12/2014 | 12/01/2022 | 2581 | male | Cazorla | Andalucia | E-Obs |
| T19 | 19/12/2014 | 17/11/2016 | 699 | female | Cazorla | Andalucia | E-Obs |
| T1C | 19/12/2014 | 06/08/2017 | 961 | female | Cazorla | Andalucia | E-Obs |
| T1J | 19/12/2014 | 10/04/2022 | 2669 | female | Cazorla | Andalucia | E-Obs |
| AM (785) | 23/06/2021 | 10/04/2022 | 292 | male | Segovia | CyL | Ornitela |
| AZ (6UC) | 10/09/2021 | 10/04/2022 | 212 | female | Segovia | CyL | Ornitela |
| AZ (1M7) | 23/06/2021 | 18/01/2022 | 210 | female | Segovia | CyL | Ornitela |
| AZ (1MM) | 23/06/2021 | 10/04/2022 | 292 | male | Segovia | CyL | Ornitela |
| AM (PCU) | 23/06/2021 | 10/04/2022 | 292 | male | Segovia | CyL | Ornitela |
| AZ (6UH) | 10/09/2021 | 06/12/2021 | 93 | female | Segovia | CyL | Ornitela |
| AZ (6UL) | 10/09/2021 | 10/04/2022 | 212 | female | Segovia | CyL | Ornitela |
| AZ (6UF) | 10/09/2021 | 10/04/2022 | 212 | female | Segovia | CyL | Ornitela |
| AZ (1M6) | 23/06/2021 | 10/04/2022 | 292 | male | Segovia | CyL | Ornitela |
| AZ (6UP) | 10/09/2021 | 10/04/2022 | 212 | female | Segovia | CyL | Ornitela |
| AZ (6UN) | 10/09/2021 | 10/04/2022 | 212 | male | Segovia | CyL | Ornitela |
| AZ (6UA) | 10/09/2021 | 09/04/2022 | 211 | female | Segovia | CyL | Ornitela |
| AZ (6P2) | 10/09/2021 | 10/04/2022 | 212 | male | Segovia | CyL | Ornitela |
| AZ (1PH) | 10/09/2021 | 10/04/2022 | 212 | male | Segovia | CyL | Ornitela |
| AZ (6UR) | 10/09/2021 | 10/04/2022 | 212 | female | Segovia | CyL | Ornitela |
| VERRUGA | 17/05/2018 | 02/04/2022 | 1416 | male | Pyrenees | Catalunya | Ornitela |
| BLASCO | 17/05/2018 | 05/08/2018 | 80 | male | Pyrenees | Catalunya | Ornitela |
| GRANIZO | 04/05/2018 | 16/08/2018 | 104 | female | Pyrenees | Catalunya | Ornitela |
| SAIA | 07/06/2019 | 09/01/2022 | 947 | male | Alto Ebro | Euskadi | Ornitela |
| PEDRA | 03/05/2018 | 09/05/2018 | 6 | male | Pyrenees | Catalunya | Ornitela |
| NOGUERA | 17/05/2018 | 10/04/2022 | 1424 | female | Pyrenees | Catalunya | Ornitela |
| TOSSAL | 17/05/2018 | 04/04/2022 | 1418 | male | Pyrenees | Catalunya | Ornitela |
| ADAN | 03/04/2019 | 10/04/2022 | 1103 | male | Pyrenees | Catalunya | Ornitela |
| QUINTANA | 17/05/2018 | 17/03/2019 | 304 | male | Pyrenees | Catalunya | Ornitela |
| COLLS | 03/04/2019 | 07/11/2021 | 948 | female | Pyrenees | Catalunya | Ornitela |
| COTOFLUIX | 17/05/2018 | 08/11/2021 | 1271 | male | Pyrenees | Catalunya | Ornitela |
| APOLO | 03/04/2019 | 10/04/2022 | 1103 | male | Pyrenees | Catalunya | Ornitela |
| PILOTO | 17/05/2018 | 02/11/2020 | 899 | male | Pyrenees | Catalunya | Ornitela |
| SANTORENS | 17/05/2018 | 10/04/2022 | 1424 | male | Pyrenees | Catalunya | Ornitela |
| ALCUDIA | 17/05/2018 | 08/04/2022 | 1422 | male | Pyrenees | Catalunya | Ornitela |
| ARTESA | 17/05/2018 | 23/06/2020 | 768 | male | Pyrenees | Catalunya | Ornitela |
| Mora-LMA | 23/02/2020 | 10/04/2022 | 777 | male | Pyrenees | Catalunya | Ornitela |
| Ter-Lomon-03R | 23/02/2020 | 10/04/2022 | 777 | female | Pyrenees | Catalunya | Ornitela |
| Capolat-N2L | 23/02/2020 | 10/04/2022 | 777 | male | Pyrenees | Catalunya | Ornitela |
| Xai-Oliana-LM3 | 23/02/2020 | 10/04/2022 | 777 | male | Pyrenees | Catalunya | Ornitela |
| Oris-Oris-N35 | 23/02/2020 | 04/05/2021 | 436 | female | Pyrenees | Catalunya | Ornitela |
| Nit-Grony-UJ4 | 23/02/2020 | 10/04/2022 | 777 | male | Pyrenees | Catalunya | Ornitela |

**Table S2.** Marginal means and 95% confidence intervals for the factors included in the best model for each model set. Abbreviations: LCI 95%= Low confidence interval, UCL 95%= Upper confidence interval.

| **Response variable** | **Predictors** | **Marginal mean** | **LCI 95%** | **UCI 95%** |
| --- | --- | --- | --- | --- |
| Monthly home-range size | Season (Winter) | 3077 | 2636 | 3518 |
|  | Season (Spring) | 5328 | 4880 | 5775 |
|  | Season (Summer) | 5273 | 4826 | 5720 |
|  | Season (Autumn) | 3281 | 2845 | 3718 |
|  | Sex (Male) | 3593 | 3177 | 4008 |
|  | Sex (Female) | 4887 | 4357 | 5417 |
|  | Breeding region (Alto Ebro) | 2552 | 2106 | 2997 |
|  | Breeding region (Segovia) | 1652 | 521 | 2783 |
|  | Breeding region (Cádiz) | 4222 | 3271 | 5174 |
|  | Breeding region (Cazorla) | 8302 | 7796 | 8808 |
|  | Breeding region (Pyrenees) | 4472 | 3771 | 5173 |
| Cumulative distance travelled per month | Season (Winter) | 892 | 685 | 1099 |
|  | Season (Spring) | 2286 | 2079 | 2493 |
|  | Season (Summer) | 2818 | 2610 | 3025 |
|  | Season (Autumn) | 1054 | 849 | 1260 |
|  | Sex (Male) | 1783 | 1580 | 1986 |
|  | Sex (Female) | 1742 | 1522 | 1961 |
|  | Breeding region (Alto Ebro) | 1325 | 1117 | 1532 |
|  | Breeding region (Segovia) | 1494 | 1131 | 1857 |
|  | Breeding region (Cádiz) | 2275 | 1965 | 2585 |
|  | Breeding region (Cazorla) | 1744 | 1534 | 1954 |
|  | Breeding region (Pyrenees) | 1974 | 1719 | 2228 |
| Monthly home-range fidelity | Season (Winter) | 0.570 | 0.545 | 0.594 |
|  | Season (Spring) | 0.673 | 0.650 | 0.694 |
|  | Season (Summer) | 0.713 | 0.692 | 0.733 |
|  | Season (Autumn) | 0.645 | 0.621 | 0.668 |
|  | Sex (Male) | 0.682 | 0.662 | 0.700 |
|  | Sex (Female) | 0.621 | 0.594 | 0.647 |
|  | Breeding region (Alto Ebro) | 0.614 | 0.585 | 0.642 |
|  | Breeding region (Segovia) | 0.761 | 0.709 | 0.805 |
|  | Breeding region (Cádiz) | 0.590 | 0.530 | 0.647 |
|  | Breeding region (Cazorla) | 0.672 | 0.638 | 0.704 |
|  | Breeding region (Pyrenees) | 0.673 | 0.629 | 0.713 |
